# Humoral immunity and infection status of PLWH following vaccination after the BA.5/BF.7 wave

**DOI:** 10.3389/fmicb.2026.1803277

**Published:** 2026-04-01

**Authors:** Yuchen Xie, Xuedong Song, Li Yang, Lijing Wang, Huixia Gao, Lixuan Zhang, Yuxin Feng, Ying Chen, Huimin Yan, Fumin Feng, Erhei Dai, Yuling Wang, Aidong Feng

**Affiliations:** 1 School of Public Health, North China University of Science and Technology, Tangshan, China; 2 Hebei Key Laboratory of Immune Mechanism of Major Infectious Diseases and New Technology of Diagnosis and Treatment, The Fifth Hospital of Shijiazhuang, North China University of Science and Technology, Shijiazhuang, Hebei, China; 3 Department of Laboratory Medicine, Handan Central Hospital, Hebei Medical University, Handan, Hebei, China; 4 Jingmen Central Hospital, Jingmen, China; 5 Hebei Coordinated Innovation Center of Occupational Health and Safety, School of Public Health, North China University of Science and Technology, Tangshan, China

**Keywords:** breakthrough infection, humoral immune, people living with HIV (PLWH), SARS-CoV-2, vaccination

## Abstract

**Objectives:**

To ensure adequate preparedness of immunocompromised populations for future pandemic waves, it is crucial to assess the humoral immune response of people living with HIV (PLWH) to existing vaccination strategies and major Omicron sublineages following BA.5/BF.7 breakthrough infection (BTI).

**Methods:**

This study enrolled 232 PLWH who had received either the CoronaVac/BBIBP-CorV or ZF2001 vaccine and experienced the BA.5/BF.7 wave in China between January and April 2023. Serum samples from each individual were collected approximately 1–6 months after the last exposure. Immunoglobulin G (IgG) and total antibodies against SARS-CoV-2 were measured using a chemiluminescent immunoassay. Neutralizing antibodies (NAbs) against D614G, BA.5, and XBB.1.5 were detected using a pseudovirus-based neutralization assay. Meanwhile, an Enterprise WeChat link was created to allow PLWH to self-report severe acute respiratory syndrome coronavirus 2 (SARS-CoV-2) infections and clinical symptoms associated with coronavirus disease 2019 (COVID-19).

**Results:**

The Omicron BA.5/BF.7 BTI rate among PLWH was 76.7%. The most commonly reported symptoms were fever, fatigue, and muscle ache. PLWH who experienced BA.5/BF.7 BTI after three doses of inactivated vaccines showed elevated levels of total antibodies, IgG, and NAb titers against D614G and BA.5. In contrast, those who experienced BTI after three doses of ZF2001 vaccines showed the highest NAb titers against D614G and BA.5. However, the neutralizing capacity of antibodies against XBB.1.5 was significantly reduced in both study cohorts. PLWH who had CD4 lymphocyte counts greater than 500 cells/μL demonstrated higher NAb titers against all tested variants and reported a lower incidence of symptoms.

**Conclusion:**

Hybrid immunity resulting from vaccination and BA.5/BF.7 BTI enhances humoral immune responses in PLWH, which may contribute to improved protection against COVID-19. However, this hybrid immunity offers limited protection against Omicron variant XBB.1.5. Regular monitoring of immune responses to new vaccines targeting emerging variants is crucial for optimizing COVID-19 prevention strategies in PLWH.

## 1 Introduction

The coronavirus disease 2019 (COVID-19), caused by severe acute respiratory syndrome coronavirus 2 (SARS-CoV-2), has resulted in a global pandemic. In December 2022, the zero-COVID policy was ended in China. According to reports from the Chinese Center for Disease Control and Prevention (Chinese CDC), between December 2022 and January 2023, SARS-CoV-2 caused a significant surge in infections, with approximately 80% of the population in China contracting SARS-CoV-2 (Fu et al., 2023). By 15 September 2024, over 776 million confirmed cases and more than seven million deaths have been reported globally since the beginning of the pandemic (World Health Organization, 2024).

With the conclusion of the zero-COVID policy in December 2022 and the subsequent surge in COVID-19 cases, there has been limited research on the immune status of PLWH following this large-scale outbreak. Indeed, vaccination remains an effective measure for preventing COVID-19 and mitigating disease severity, particularly for PLWH (Wang and Lai, 2024; Rahmani et al., 2022; Xueying et al., 2022). However, compared to individuals without HIV, PLWH face nearly a 30% higher risk of hospitalization and mortality following COVID-19 infection (Braunstein et al., 2023). Furthermore, among PLWH, factors such as low CD4 lymphocyte counts and high viral loads are significant risk factors associated with COVID-19 mortality (Drobyshevskaya et al., 2023; Oprea et al., 2024). According to the World Health Organization (WHO), the continuous evolution of SARS-CoV-2 has resulted in increased immune evasion from vaccines and vaccination-induced immunity, particularly with emerging Omicron subvariants such as XBB.1.5, XBB.1.16, EG.5.1, and BA.2.86. By May 2023, the Omicron XBB subvariant had become the most prevalent strain (World Health Organization, 2023). Studies have demonstrated that even in the general population, resistance to new variants has developed, with significantly reduced neutralization activity (Zhao et al., 2024a, b; Wang et al., 2023). Therefore, it is critical to evaluate the humoral immune response to existing vaccination strategies and major Omicron sublineages, particularly after BA.5/BF.7 breakthrough infections in PLWH.

In light of the ongoing evolution of COVID-19, this study critically examined the prevalence and severity of the virus among PLWH following the end of the zero-COVID policy. Furthermore, the study explored the protective role of antibodies within this vulnerable population. The findings emphasize the importance of these factors in shaping effective COVID-19 vaccination strategies and implementing targeted preventive measures for PLWH, ultimately enhancing their health outcomes.

## 2 Methods

### 2.1 Study design, participants, and sample collection

**Figure 1:**
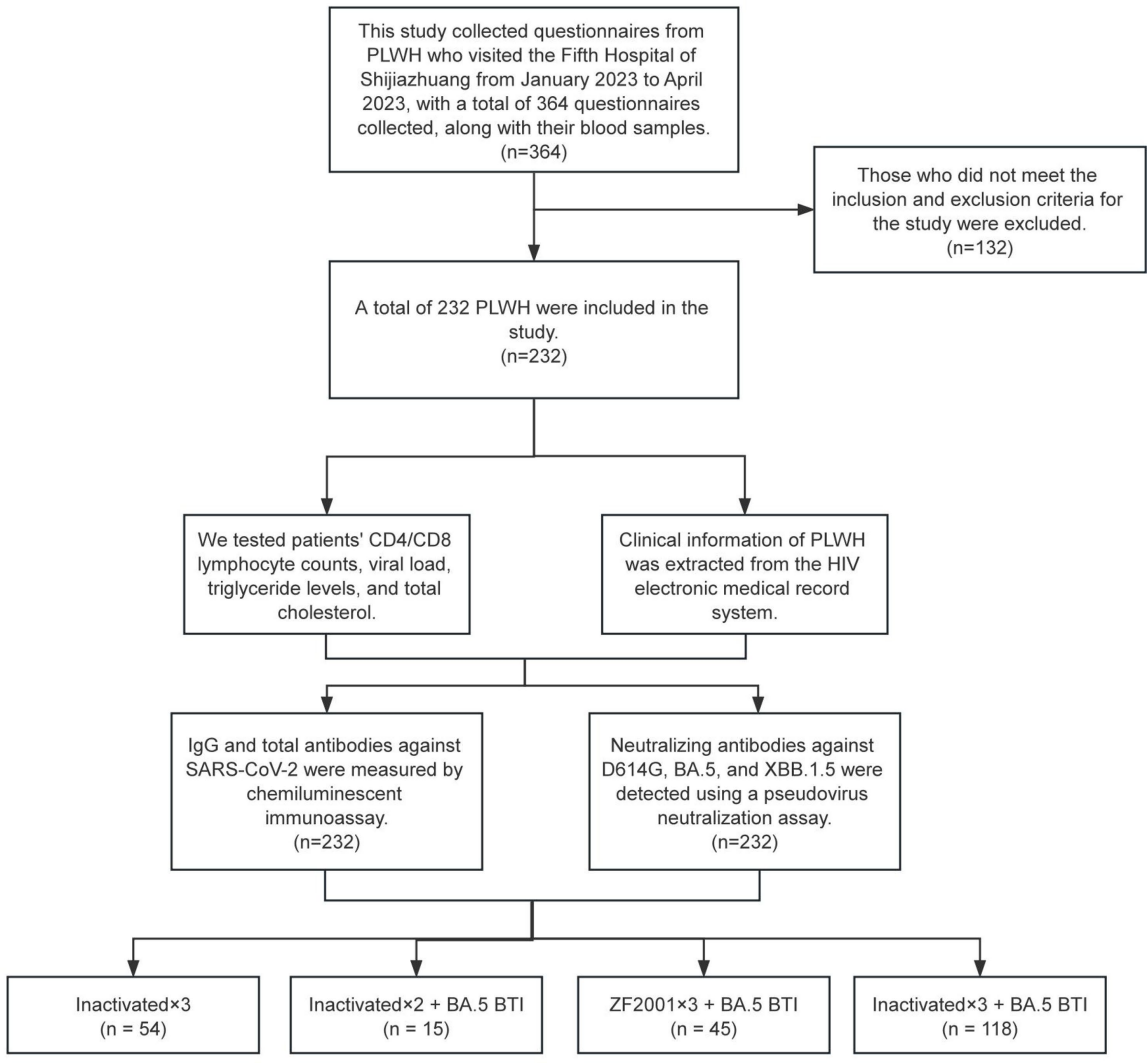
Study flowchart.

This study collected questionnaires and blood samples from PLWH who visited the Fifth Hospital of Shijiazhuang (provincial-level infectious disease hospital) between January and April 2023. Participants were vaccinated with either CoronaVac/BBIBP-CorV or ZF2001 and experienced the Omicron BA.5/BF.7 wave. An Enterprise WeChat link was created to enable PLWH to self-report SARS-CoV-2 infections and COVID-19 symptoms. Participants were asked to report SARS-CoV-2 infection confirmed by antigen testing or nucleic acid (PCR) testing (Supplementary Table S3). The survey mainly includes baseline information, SARS-CoV-2 infection status, and clinical symptoms after infection. A total of 364 questionnaires were collected. The inclusion criteria were as follows: (1) PLWH who had received SARS-CoV-2 vaccination; (2) availability of complete questionnaire information; and (3) signed informed consent. After enrollment and data collection, the participants were grouped retrospectively based on their vaccination regimen and breakthrough infection (BTI) status. Individuals who had received two vaccine doses but did not report BTI were excluded from further analysis due to a small sample size. A total of 232 PLWH were included in the study. The study selection process is shown in Figure 1. PLWH were categorized into four cohorts: The first cohort included PLWH who received three doses of inactivated CoronaVac/BBIBP-CorV but did not experience SARS-CoV-2 breakthrough infections (Inactivated×3 Cohort); the second cohort included PLWH who completed two doses of inactivated CoronaVac/BBIBP-CorV and reported Omicron BA.5 breakthrough infections (Inactivated×2 + BA.5 BTI Cohort); the third cohort included PLWH who completed three doses of ZF2001 and reported BA.5 breakthrough infections (ZF2001 × 3 + BA.5 BTI Cohort); and the fourth cohort included PLWH who received homologous booster doses of inactivated CoronaVac/BBIBP-CorV and reported BA.5 breakthrough infections (Inactivated×3 + BA.5 BTI Cohort).

Each participant provided a 5 mL blood sample for testing and serum isolation. Serum samples were collected approximately 1–6 months after the last exposure. CD4/CD8 lymphocyte counts, viral load, triglyceride levels, and total cholesterol were measured among PLWH. Sera were isolated by centrifugation at 3000 rpm for 10 min and stored at −80 °C until testing. Clinical data were obtained from the electronic medical records of PLWH, including age, gender, BMI, WHO staging system for HIV infection, and existing treatment regimens. Informed consent was obtained from all participants. The study protocol was conducted in accordance with the Declaration of Helsinki and approved by the Medical Ethics Committee of the Fifth Hospital of Shijiazhuang.

### 2.2 Reliability and validity analysis of the questionnaire survey

To ensure the reliability and validity of the questionnaire used to assess recent SARS-CoV-2 infection, a series of psychometric tests were conducted. The internal consistency was evaluated using Cronbach’s *α* = 0.69 (Table 1), indicating acceptable reliability. The Kaiser–Meyer–Olkin (KMO) measure of sampling adequacy was 0.75, and the Bartlett’s test of sphericity was significant (*p* < 0.0001), supporting the suitability of the questionnaire for capturing infection status (Table 2). Additionally, the questionnaire was pilot-tested in a small group of PLWH to verify clarity and the accuracy of self-reported infection data.

**Table 1:** Reliability analysis of measurement dimensions.

Cronbach’s alpha	Number of items
0.690	21

**Table 2:** Validity analysis.

**KMO and Bartlett’s Test**		
KMO sampling adequacy measure		0.751
Bartlett’s sphericity test	Approximate chi-square	1763.567
	Degrees of freedom	210
	*P*-value	<0.0001

### 2.3 Total antibodies and IgG by chemiluminescent microparticle immunoassay (CLIA)

Magnetic particle chemiluminescence was used to detect 2019-nCoV IgG and 2019-nCoV Ab following the manufacturer’s protocol. The samples were thawed in a refrigerator at 4 °C the day before testing and then kept at room temperature with the reagents until analysis. The serum was mixed in a pipette, and 200 μL of the mixed serum was added to the reaction cup. Serum levels of 2019-nCoV Ab were measured using an automated chemiluminescence immunoassay analyzer (Caris200) with the accompanying kits (Innodx, Xiamen, China) following the manufacturer’s instructions. The 2019-nCoV antibody test result was negative when the sample-to-cutoff (S/CO) value was less than 1.0 COI. Serum levels of 2019-nCoV IgG were measured using an automated chemiluminescence immunoassay analyzer (YHLO iFlash 3,000) with the 2019-CoV IgG antibody detection kit (YHLO, Shenzhen, China) following the manufacturer’s instructions. The antibody levels <10 AU/mL were considered non-responsive.

### 2.4 Cell lines

Human embryonic kidney HEK-293 T cells were cultured at 37 °C and 5% CO_2_ in Dulbecco’s modified Eagle’s medium (DMEM, Gibco, USA) supplemented with 10% (v/v) heat-inactivated fetal bovine serum (FBS, Gibco, USA) and 1% penicillin–streptomycin (Gibco, USA). The cells were disrupted at confluence with 0.25% trypsin in 1 mM EDTA (Solarbio, China) every 48–72 h.

### 2.5 Spike plasmid pseudovirus production

Pseudovirus particles were generated as previously described by cotransfecting HEK-293 T cells (ATCC, CRL-3216) with human immunodeficiency virus backbones expressing firefly luciferase (pNL4-3-R-E-luciferase) and the pcDNA3.1 vector encoding the S protein of the D614G, BA.5, and XBB.1.5 plasmids. Codon-optimized, full-length open reading frames of the spike genes of the D614G, BA.5, and XBB.1.5 strains were synthesized by GenScript (Nanjing, China). The medium was replaced with fresh medium at 24 h, and the supernatants were harvested at 48 h post-transfection and clarified by centrifugation at 300 × g for 10 min, aliquoted, and stored at −80 °C until use.

### 2.6 Pseudovirus neutralization assay

A SARS-CoV-2 pseudovirus neutralization assay (pVNT) was performed (Zhu et al., 2023) using HeLa overexpressing hACE2 orthologs. All viruses were titrated to normalize the viral input between assays. Duplicate 3-fold, 8-point serial dilutions of heat-inactivated sera (starting at 1:30) were incubated with 500–1,000 TCID50 of the SARS-CoV-2 pseudotyped virus for 1 h at 37 °C and 5% CO_2_. Subsequently, 1 × 10 (Rahmani et al., 2022) HeLa-ACE2 cells were added to each well and incubated at 37 °C and 5% CO_2_ for 48 h. After incubation, the supernatant was removed, and the cells were lysed using a passive lysis buffer (Vazyme) for 3 min at room temperature. The lysates were transferred to an opaque white 96-well plate, reconstituted by adding luciferase assay buffer (Vazyme), and the proteins were mixed with each lysate. Luminescence was measured immediately after mixing using a GloMax 96 Microplate Luminometer (Promega). The neutralization titer (NT_50_) was determined by luciferase activity using a four-parameter non-linear regression inhibitor curve in GraphPad Prism 9.5.1 (GraphPad Software). NT_50_ was reported as the reciprocal serum dilution causing a 50% reduction in relative light units. A sample with an NT_50_ value of ≤30 (the detectable limit) was considered negative for neutralizing antibodies (NAbs) and assigned a nominal value of 10 in geometric mean titer (GMT) calculations, representing the lowest serum dilution MMfactor used in the assay.

### 2.7 Statistical analysis

All statistical analyses were performed using GraphPad Prism 9.5.1 (GraphPad Software Inc., CA, USA) and SPSS 26.0 (SPSS Inc., IBM, New York, United States). The data distribution normality was assessed using the Shapiro–Wilk test. Continuous variables were expressed as medians and interquartile ranges [IQR]. Categorical data were reported as *n* (%), and comparisons were analyzed by the chi-squared test or Fisher’s exact test. The Wilcoxon rank-sum test or Mann–Whitney *U* test was used for comparison between two groups. The Kruskal–Wallis test with the false discovery rate method was used for multiple comparisons were appreciated. Multiple comparisons were performed using Tukey’s test. All samples were tested using two-sided tests, and *p* < 0.05 was considered statistically significant.

## 3 Results

### 3.1 Demographic characteristics and symptom frequency distribution of SARS-CoV-2 infection among PLWH

This cross-sectional study, conducted from January to April 2023, included 232 PLWH who experienced the BA.5/BF.7 wave and received at least two doses of CoronaVac/BBIBP-CorV or ZF2001 SARS-CoV-2 vaccines. The median age of the participants was 41 (40–45) years, with men comprising 94.8% (*n* = 220) of the cohort. Among the PLWH, 26 had underlying health conditions, with hypertension (11.2%) and diabetes (5.2%) being the most common comorbidities. A total of 118 participants (50.9%) received booster vaccines (Inactivated×2 Cohort + BA.5 BTI Cohort), all of which were homologous vaccinations. Clinical and laboratory data of the PLWH are presented in Supplementary Table S1.

The study utilized questionnaires for self-reported clinical symptoms of SARS-CoV-2 infection among PLWH. Based on our data, the estimated peak of the Omicron BA.5/BF.7 wave occurred in January 2023 (Supplementary Figure S1). Among the participants, 178 (76.7%) experienced Omicron BA.5 breakthrough infections, with 152 (85.4%) reporting symptoms. The symptom incidence rates for each group are shown in Table 3 (Inactivated×3 + BA.5 BTI vs. ZF2001 × 3 + BA.5 BTI vs. Inactivated×3 + BA.5 BTI: 86.6% vs. 88.9% vs. 83.9%), with patients who received two doses of inactivated vaccine and three doses of ZF2001 vaccine having a higher symptom incidence rate compared to those who received three doses of inactivated vaccine. The most common symptoms were fever (Inactivated×2 + BA.5 BTI vs. ZF2001 × 3 + BA.5 BTI vs. Inactivated×3 + BA.5 BTI: 33.3% vs. 55.6% vs. 55.1%), fatigue (Inactivated×2 + BA.5 BTI vs. ZF2001 × 3 + BA.5 BTI vs. Inactivated×3 + BA.5 BTI: 46.7% vs. 28.9% vs. 29.7%), and muscle ache (Inactivated×2 + BA.5 BTI vs. ZF2001 × 3 + BA.5 BTI vs. Inactivated×3 + BA.5 BTI: 46.7% vs. 22.2% vs. 25.4%) (Table 3).

**Table 3:** Demographic characteristics of the PLWH.

**Characteristics**	**Inactivated × 3**	**Inactivated × 2 + BA.5 BTI**	**ZF2001 × 3 + BA.5 BTI**	**Inactivated × 3 + BA.5 BTI**
No. of participants (*n*, %)	54 (23.3)	15 (6.5)	45 (19.4)	118 (50.9)
Age (Median, IQR)	42 (35, 54)	35 (32, 53)	41 (33, 55)	40 (33, 52)
Gender (*n*, %)				
Male	50 (92.6)	15 (100)	43 (95.6)	112 (94.9)
Female	4 (7.4)	0 (0.0)	2 (4.4)	6 (5.1)
BMI (Median, IQR)	22.6 (16.1, 44.8)	21.4 (19.5, 23.1)	21.6 (20.0, 24.0)	22.0 (16.7, 35.5)
Underlying diseases (*n*, %)				
Yes	4 (7.4)	2 (7.7)	11 (24.4)	9 (7.6)
Hypertension	NA	1 (6.7)	5 (11.1)	6 (5.1)
Diabetes	1 (1.9)	NA	4 (8.9)	4 (3.4)
Chronic hepatitis	2 (3.7)	NA	1 (2.2)	1 (0.8)
Hyperlipidemia	1 (1.9)	2 (13.3)	2 (4.4)	NA
Omicron BA.5 symptomatic infection (*n*, %)				
Yes	NA	13 (86.6)	40 (88.9)	99 (83.9)
Fever	NA	5 (33.3)	25 (55.6)	65 (55.1)
Fatigue	NA	7 (46.7)	13 (28.9)	35 (29.7)
Muscle aches	NA	7 (46.7)	10 (22.2)	30 (25.4)
Headache	NA	4 (26.7)	10 (22.2)	27 (22.9)
Taste and/or smell loss	NA	4 (26.7)	4 (8.9)	12 (60.0)
Sore throat	NA	3 (20.0)	8 (7.8)	28 (23.7)
Cough	NA	3 (20.2)	12 (26.7)	36 (30.5)
Expectoration	NA	3 (20.2)	4 (8.9)	10 (8.5)
Nasal congestion	NA	2 (13.3)	4 (8.9)	23 (19.5)
Runny nose	NA	2 (13.3)	6 (13.3)	17 (14.4)
Dyspnea	NA	2 (13.3)	1 (2.2)	3 (2.5)
Diarrhea	NA	0 (0.0)	1 (2.2)	7 (5.9)
Emesis	NA	0 (0.0)	0 (0.0)	2 (1.7)
Others	NA	0 (0.0)	0 (0.0)	1 (0.8)
Treatment methods (*n*, %)				
Hospitalization	NA	0 (0.0)	0 (0.0)	1 (0.8)
Outpatient or emergency	NA	2 (13.3)	1 (0.8)	7 (5.9)
Home isolation	NA	13 (86.7)	44 (97.8)	110 (93.2)
Sampling time (Median, IQR)				
Sampling distance time for breakthrough infection	NA	98 (83, 106)	94 (75, 108)	96 (67, 110)
Days between booster vaccination and follow-up	459 (369, 593)	603 (53, 641)	598 (566, 621)	447 (389, 467)

IQR, Interquartile range; BMI, Body mass index; NA, Not available; “Others” represents the occurrence of symptoms not shown in the table.

### 3.2 Responses of total antibody and IgG levels against SARS-CoV-2 among PLWH

**Figure 2:**
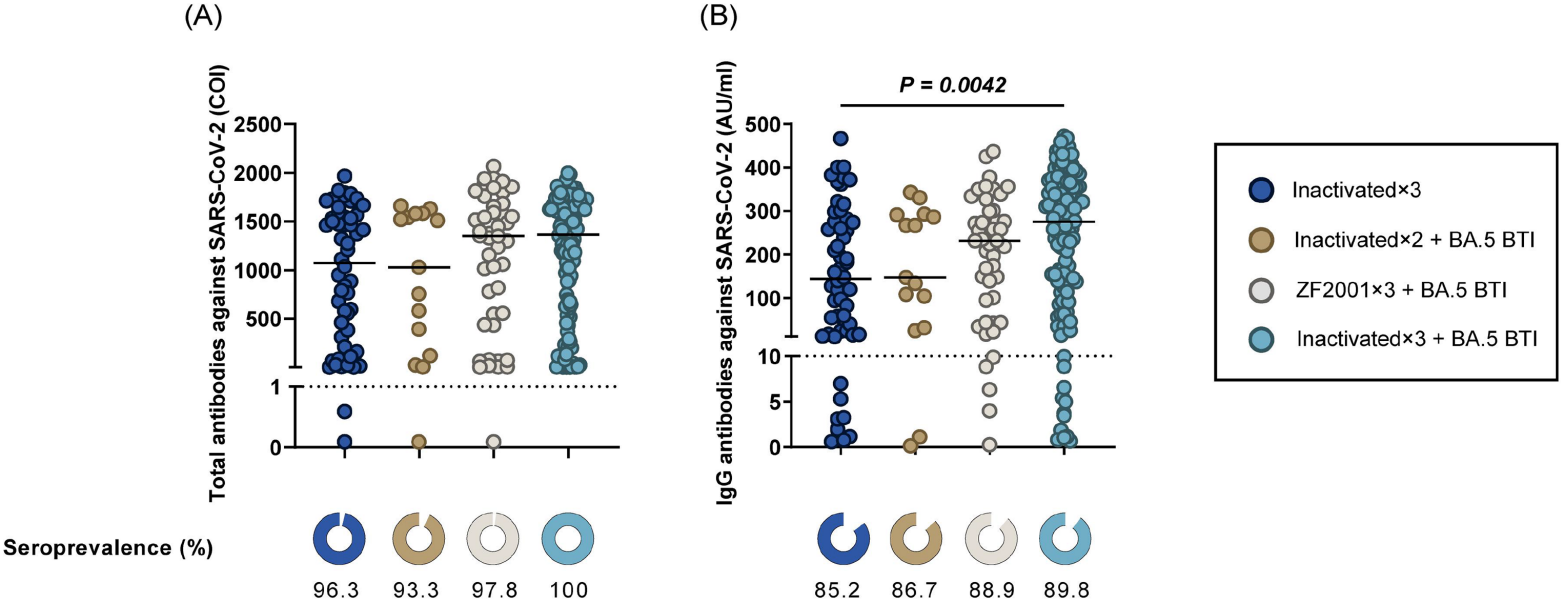
The responses of total antibody and IgG antibody against SARS-CoV-2 among PLWH. **(A)** Total antibody responses at inactivated×3 cohort, inactivated×2 + BA.5 BTI cohort, ZF2001 × 3 + BA.5 BTI cohort, and inactivated×3 + BA.5 BTI cohort among the 232 PLWH. **(B)** IgG antibody responses at inactivated×3 cohort, inactivated×2 + BA.5 BTI cohort, ZF2001 × 3 + BA.5 BTI cohort, and inactivated×3 + BA.5 BTI cohort among the 232 PLWH. All values are presented as the median and interquartile range. The black dotted line represents the positivity cut-off for neutralizing antibodies (total antibody: <10 AU/mL is negative; IgG: S/CO < 1.0 COI is negative). IgG, immunoglobulin G.

The total antibodies and IgG against SARS-CoV-2 were measured using a magnetic particle chemiluminescence method. For comparative analysis, PLWH were categorized into Inactivated × 3 Cohort, Inactivated × 2 + BA.5 BTI Cohort, ZF2001 × 3 + BA.5 BTI Cohort, and Inactivated × 3 + BA.5 BTI Cohort based on vaccination and COVID-19 infection status. In the study, we observed that the seropositivity rate for SARS-CoV-2 total antibodies ranged from 93.3 to 100%, while the IgG seropositivity rate ranged from 85.2 to 89.8%. The Inactivated×3 + BA.5 BTI Cohort exhibited comparatively elevated total antibody and IgG levels. Additionally, the ZF2001 × 3 + BA.5 BTI Cohort and Inactivated × 3 + BA.5 BTI Cohort exhibited higher total antibody and IgG levels than the Inactivated × 3 Cohort and Inactivated × 2 + BA.5 BTI Cohort. A statistically significant difference in IgG concentrations was observed between the Inactivated×3 Cohort and Inactivated × 3 + BA.5 BTI Cohort (*p* < 0.05) (Figure 2).

### 3.3 Neutralizing capacity against different variants of SARS-CoV-2 among PLWH

**Figure 3:**
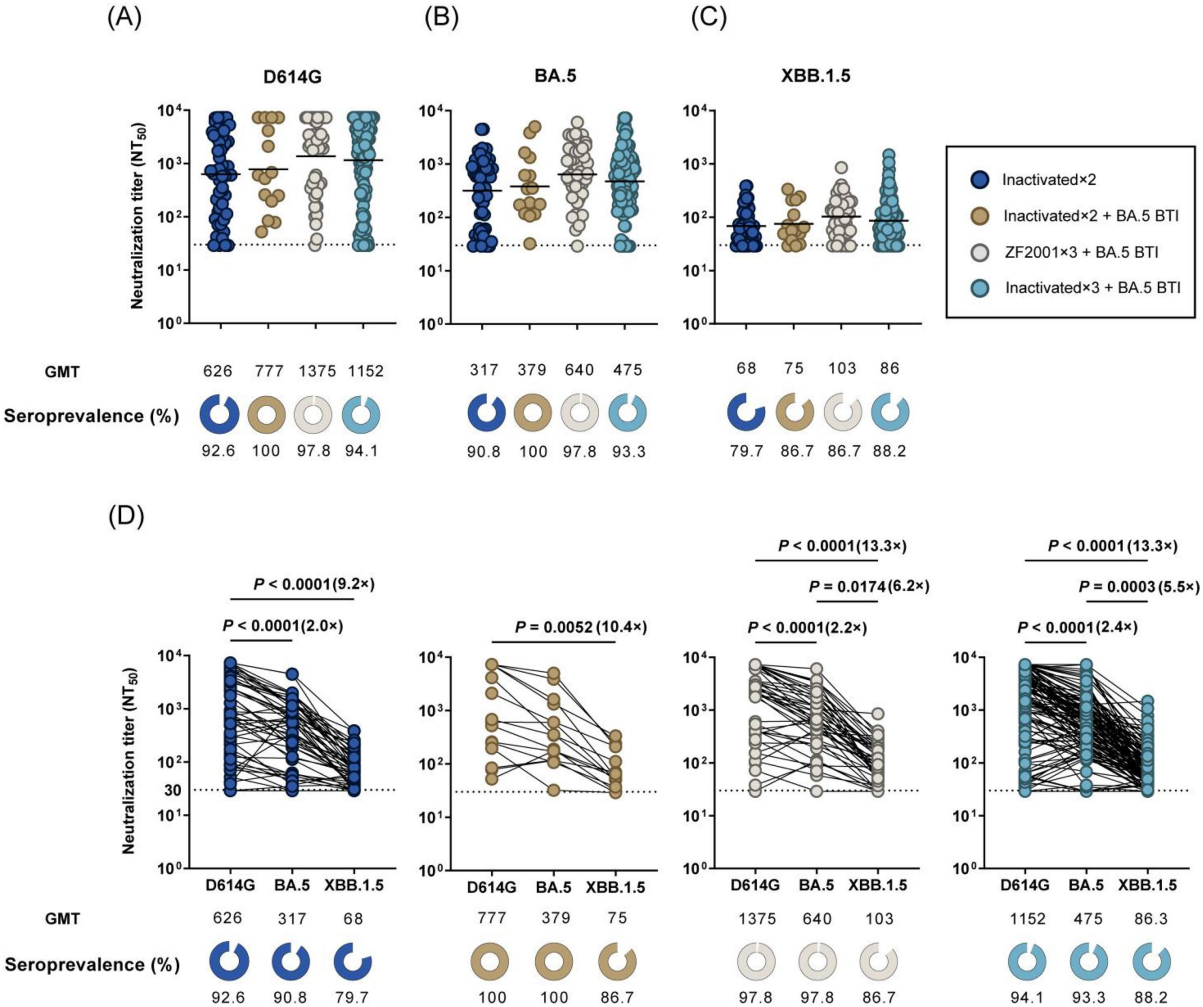
NAb titers against SARS-CoV-2 variants. **(A)** Comparison of NAb titers against D614G among different Omicron infection statuses and vaccine doses. **(B)** Comparison of NAb titers against Omicron BA.5 among different Omicron infection statuses and vaccine doses. **(C)** Comparison of NAb titers against Omicron XBB.1.5 among different Omicron infection statuses and vaccine doses. **(D)** Comparison of virus-specific NAb titers against D614G and Omicron BA.5, XBB.1.5 subvariants at Inactivated×3 Cohort, Inactivated ×2 + BA.5 BTI Cohort, ZF2001 × 3 + BA.5 BTI Cohort, and Inactivated×3 + BA.5 BTI Cohort. The black dotted line represents the positivity cut-off for neutralizing antibodies (NT_50_ of 30). The GMTs are shown under each column along with the percentage of individuals with NT_50_ values above 30. The fold change of GMT is also denoted after the *p*-value.

The NAbs in PLWH were analyzed using a pseudovirus neutralization assay. NAb titers against the ancestral D614G strain and Omicron BA.5 and XBB.1.5 variants were evaluated across the four PLWH groups. The ZF2001 × 3 + BA.5 BTI Cohort and Inactivated×3 + BA.5 BTI Cohort demonstrated higher neutralization capacities against D614G, BA.5, and XBB.1.5 variants than the Inactivated × 2 Cohort and Inactivated×2 + BA.5 BTI Cohort (Figures 3A–C). Across all cohorts, titers against D614G were the highest, while titers against BA.5 and XBB.1.5 were significantly lower, with XBB.1.5 showing the lowest neutralization rates (79.7–86.7% positivity across all cohorts).

Specifically, all individuals in the Inactivated × 2 + BA.5 BTI Cohort exhibited NAbs against the D614G and BA.5 strains, while 13.3% could not neutralize the XBB.1.5 variant. The geometric mean titers (GMT) for BA.5 and XBB.1.5 were 2.0–10.4 times lower than D614G. Furthermore, the GMT for XBB.1.5 was significantly lower than that for D614G (*p* < 0.05, Figure 3D). For patients in the ZF2001 × 3 + BA.5 BTI Cohort, NAbs were detectable against all tested variants in 86.7–97.8% of individuals. Compared to D614G, GMTs for BA.5 and XBB.1.5 were reduced by 2.1–13.3 fold. Additionally, the ZF2001 × 3 + BA.5 BTI Cohort demonstrated the strongest neutralization capacity against all tested strains compared to other cohorts (Figure 3D). In the Inactivated × 3 + BA.5 BTI cohort, NAbs were detected in 88.2–94.1% of individuals. The GMTs for BA.5 and XBB.1.5 were 2.4–13.3 times lower than those for D614G (Figure 3D). Finally, the Inactivated×3 cohort exhibited the weakest neutralization capacity against all tested variants, with NAbs detected in 79.7–92.6% of individuals. The GMTs for BA.5 and XBB.1.5 were 3.0–9.2 times lower than those for D614G (Figure 3D). Interestingly, the GMTs for D614G in the Inactivated×2 cohort, ZF2001 × 3 + BA.5 BTI cohort, and Inactivated×3 + BA.5 BTI cohort were significantly higher than those for BA.5 and XBB.1.5 (*p* < 0.0001). Furthermore, in the ZF2001 × 3 + BA.5 BTI cohort and Inactivated×3 + BA.5 BTI cohort, the GMTs for XBB.1.5 were significantly lower than those for BA.5 (*p* < 0.05).

### 3.4 Risk factors associated with NAb titers among PLWH

**Figure 4:**
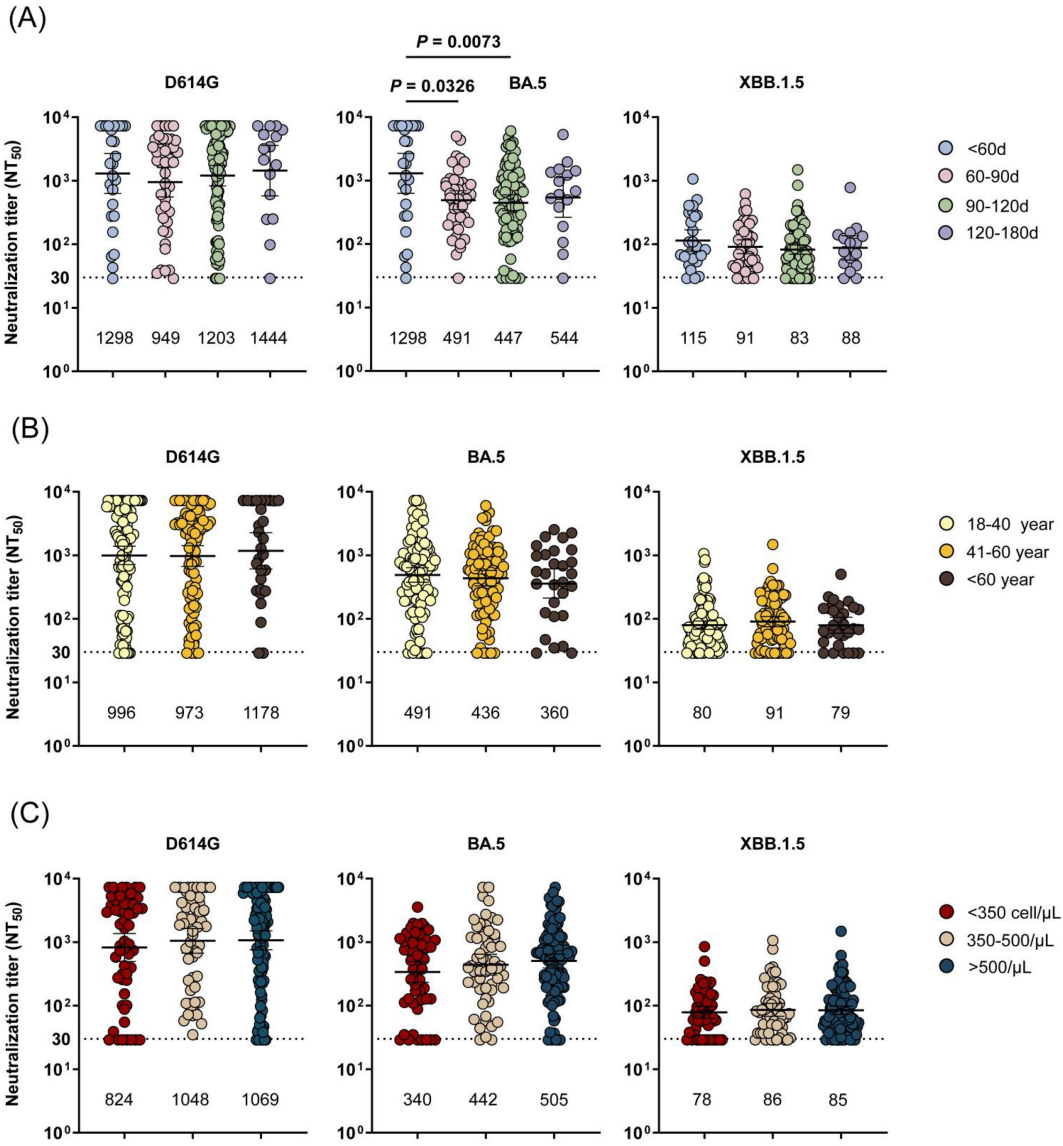
Comparison of NAb titers against SARS-CoV-2 variants of PLWH by risk factors. **(A)** Comparison of neutralizing antibody (NAb) titers against SARS-CoV-2 variants of PLWH at different times from infection to sampling. **(B)** Comparison of neutralizing antibody (NAb) titers against SARS-CoV-2 variants of PLWH at different ages. **(C)** Comparison of neutralizing antibody (NAb) titers against SARS-CoV-2 variants of PLWH at different CD4 lymphocyte counts. The black dotted line represents the positivity cut-off for neutralizing antibodies (NT_50_ of 30). The GMTs are shown under each column.

According to the ‘Expert Consensus on the Clinical Diagnosis and Treatment of COVID-19 in human immunodeficiency virus infection/acquired immunodeficiency (HIV/AIDS) Patients’ and ‘Chinese guidelines for diagnosis and treatment of HIV/AIDS (2024 edition),’ advanced age and CD4 cell count are important factors for the poor prognosis of COVID-19 infection in HIV/AIDS patients. At the same time, antibody levels tend to decrease over time. Therefore, we conducted further analyses to determine whether the time from infection to sampling, age groups, and CD4 lymphocyte count were associated with NAb titers among PLWH, as well as to assess BTI rates and symptom incidence (Figure 4 and Supplementary Table S2). The impact of the time from infection to sampling, age groups, and CD4 lymphocyte counts on the total antibody and IgG levels was evaluated, but no significant differences were found. Therefore, these factors were not mentioned in this study.

Patients were categorized based on the time from infection to sampling: <60 days, 60–90 days, 90–120 days, and 120–180 days. After Omicron BA.5 breakthrough infection, the NAb titers against all tested SARS-CoV-2 variants were comparable across different time intervals, except for PLWH sampled <60 days post-infection. Notably, we found that NAb titers against each variant reached relatively high levels within <60 days post-infection. However, over time, the NAb titers against the BA.5 variant decreased significantly from their peak, indicating that the protective effect of antibodies diminishes over time and thereby increases the risk of reinfection. However, for the original strain D614G, the majority of individuals maintain a certain neutralization potency (Figure 4A). Specifically, PLWH sampled within <60 days post-infection exhibited significantly higher NAb titers compared to those sampled at 60–90 days and 90–120 days post-infection (*p* < 0.05).

To examine the impact of age on NAb titers, patients were categorized into three cohorts: young (18–40 years), middle-aged (41–60 years), and elderly (>60 years). Younger individuals exhibited slightly higher NAb titers against the BA.5 variant. Overall, the impact of age on NAb titers is not significant. Interestingly, elderly PLWH demonstrated lower infection and symptom incidence rates (Figure 4B and Supplementary Table S2).

Finally, the effect of different CD4 counts on NAb titers was assessed. The findings indicated that NAbs titers against all variants increased progressively with higher CD4 counts, and PLWH with CD4 lymphocyte counts greater than 500 cells/μL demonstrated higher NAb titers against all tested variants and experienced a lower incidence of symptoms (Figure 4C and Supplementary Table S2).

## 4 Discussion

In this study, a cross-sectional analysis was conducted to elucidate the epidemiological characteristics and antibody dynamics of PLWH who had been vaccinated prior to experiencing the Omicron BA.5/BF.7 wave in China. Based on self-reported COVID-19 infection data collected through questionnaires, 178 (76.7%) patients experienced Omicron BA.5 breakthrough infections, while 57 (59.3%) of these individuals reporting symptoms. PLWH who received three doses of either the CoronaVac/BBIBP-CorV or ZF2001 vaccine and subsequently experienced breakthrough infections exhibited elevated levels of total antibodies, IgG, and NAb titers. However, the XBB.1.5 variant demonstrated a higher level of resistance.

During the Omicron BA.5/BF.7 wave in China, the overall infection rate was reported to be 84.5% following vaccination (Hu et al., 2023), with healthcare workers experiencing an infection rate of 93.7% (Song et al., 2024). In this study, the BTI rate among PLWH was 76.7%, which was lower than other populations. This lower infection rate may be attributed to the heightened awareness and concern about COVID-19 among PLWH, leading to greater adherence to vaccination and public health measures, thereby reducing infection risk. Additionally, some studies have suggested that antiretroviral therapy (ART) administered to PLWH may have a suppressive effect on the replication of SARS-CoV-2 (Fintelman-Rodrigues et al., 2020; Qi et al., 2023), which could be another contributing factor to the lower infection rate. However, these explanations remain speculative, as the present study was not designed to evaluate the impact of ART on SARS-CoV-2 infection risk. Further studies are required to formally assess this hypothesis.

Similar to symptoms reported in the general population (Hu et al., 2023; Song et al., 2024; Grant et al., 2020), the most commonly observed symptoms among PLWH were fever and fatigue, only one PLWH in this study reported hospitalization during infection, indicating a relatively mild disease severity. Vaccination and subsequent breakthrough infections played a protective role among PLWH, a hypothesis further supported by antibody testing. PLWH who experienced breakthrough infections after receiving booster vaccinations exhibited significantly higher levels of total antibodies, IgG, and NAb titers compared to other populations. Moreover, the PLWH included in this study were in stable health conditions, which likely contributed to the lower severity of COVID-19 observed in this population.

Higher levels and increased avidity of antibodies are associated with a lower probability of reinfection and milder disease upon reinfection in COVID-19 patients. The persistence and maturation of these antibodies play a crucial role in long-term immunity (Ali et al., 2021; Löfström et al., 2021). This study demonstrates that PLWH who experienced breakthrough infections after receiving three doses of either the CoronaVac/BBIBP-CorV or ZF2001 vaccine exhibited enhanced levels of total antibodies, IgG, and NAb titers against all tested variants. In particular, PLWH who received three doses of the inactivated vaccine and experienced breakthrough infections showed a significant increase in total antibodies, IgG, and neutralizing antibodies compared to those who received only three doses of the inactivated vaccine. However, there were no statistically significant differences in total antibodies, IgG, and neutralizing antibody levels between patients who received three doses of the inactivated vaccine and those who received two doses followed by a breakthrough infection. This indicates that the hybrid immunity formed through booster vaccination and BTI plays a crucial role in preventing SARS-CoV-2 infections. However, the NAb titers against the Omicron XBB.1.5 variant in PLWH who experienced breakthrough infections remained at low levels, consistent with previous studies (Zhao et al., 2024a, b; Wang et al., 2023). This suggests that even with hybrid immunity, the existing NAbs may not provide sufficient protection against emerging variants such as XBB.1.5, leaving PLWH vulnerable to reinfection. Based on these findings, PLWH should receive updated vaccines specifically targeting XBB.1.5 or other newly emerging SARS-CoV-2 variants to strengthen their immunity. This proactive approach could help mitigate the adverse effects of reinfection on HIV management and overall health outcomes in this population.

Studies have shown that NAb titers gradually decline over time (Ishizaki et al., 2022; Takahashi et al., 2023). Our results similarly indicate that within 60 days post-infection, NAbs against the BA.5 variant exhibited higher neutralizing activity, which significantly decreased between 60 and 180 days. This highlights the necessity for regular vaccination to maintain immune protection. Notably, while this study focused on homologous vaccination, PLWH vaccinated with ZF2001 showed slightly higher NAb titers compared to those vaccinated with CoronaVac/BBIBP-CorV. Furthermore, this study found that age had little impact on NAb levels. Interestingly, the findings revealed that elderly PLWH had lower infection and symptom incidence rates, which contrasts with the results of studies by Zhao X-J et al. (Zhao et al., 2024a, b; Wünsch et al., 2022; Niu et al., 2020). While some studies suggest that elderly individuals experience lower symptom incidence, they often face more severe disease and prolonged recovery (Damanti et al., 2023; Bicalho et al., 2024). This study hypothesized that hybrid immunity might mitigate differences in vaccine response and age-related outcomes. Finally, PLWH were categorized into three groups based on CD4 lymphocyte cell counts. Furthermore, this analysis revealed that PLWH with CD4 < 200 cells/μL had significantly lower NAb titers, and NAb titers increased progressively with higher CD4 lymphocyte cell counts. This underscores the critical role of CD4 lymphocyte cell counts in maintaining immune competence among PLWH. Overall, the antibody response in PLWH may be influenced by various factors, including time since infection and CD4 lymphocyte cell counts. These findings emphasize the need to consider multiple aspects of PLWH health when developing strategies for preventing and managing COVID-19.

The study has several limitations. First, this is a cross-sectional study, which limits the ability to monitor disease progression and dynamically evaluate antibody titers over time. The persistence of antibodies against Omicron and its subvariants requires more longitudinal data to be fully understood. Second, this study included a limited number of elderly PLWH patients and severe cases, which may have restricted the robustness of the assessments based on the impact of age and disease severity on antibodies. Future studies could expand the sample size to include more participants across different age groups and severe cases of PLWH for a more comprehensive assessment. Third, this study did not evaluate cellular immunity, which is a fundamental criterion for determining disease severity. Understanding the diverse and critical roles of cellular immunity can enable a more in-depth evaluation of different populations. Finally, although participants were asked to report infections confirmed by antigen or nucleic acid testing, reliance on self-reported information may have introduced potential misclassification.

## 5 Conclusion

Hybrid immunity generated by vaccination and breakthrough infections, which can stimulate humoral immune responses, was associated with a trend toward lower incidence and milder symptoms of COVID-19 breakthrough infections in PLWH. However, for Omicron variants, such as XBB.1.5, which exhibit significant immune evasion characteristics, existing vaccines and infection-induced neutralizing antibodies provide limited protection for PLWH, making them vulnerable to reinfection. Therefore, vaccination strategies need to be updated to adapt to the evolution of viral strains, preventing SARS-CoV-2 reinfection and safeguarding PLWH health. The absence of cellular immune data and the variability in the intervals between vaccination, infection, and sample collection limit our ability to comprehensively characterize the immune landscape induced by vaccination and breakthrough infections. Future studies incorporating T-cell immunity may help provide a more complete understanding of the correlates of protection in this population.

## Data Availability

The raw data supporting the conclusions of this article will be made available by the authors, without undue reservation.

## Supplementary material

SUPPLEMENTARY FIGURE S1 The prevalence and peak occurrence time of the Omicron BA.5/BF.7 wave.
