# Supplementary material for: Humoral immunity and infection status of PLWH following vaccination after the BA.5/BF.7 wave
Source: Front Microbiol. 2026 Apr 1;17:1803277. doi: 10.3389/fmicb.2026.1803277 (PMC13079649; doi:10.3389/fmicb.2026.1803277)
Supplement: Supplementary file 1 [file Table_1.DOCX]

**Table S1. Clinical and laboratory Information of PLWH.**

| **Characteristics** | **Inactivated×3** | **Inactivated×2 + BA.5 BTI** | **ZF2001×3 + BA.5 BTI** | **Inactivated×3 + BA.5 BTI** |
| --- | --- | --- | --- | --- |
| **WHO disease staging system (n, %)** |  |  |  |  |
| Stage I | 38 (70.3) | 13 (86.6) | 29 (64.4) | 91 (77.1) |
| Stage II | 2 (3.7) | 0 (0.0) | 0 (0.0) | 1 (0.8) |
| Stage III | 3 (5.5) | 1 (6.6) | 2 (4.4) | 3 (2.5) |
| Stage IV | 11 (20.3) | 1 (6.6) | 14 (31.1) | 23 (19.4) |
| **Treatment Plan (n, %)** |  |  |  |  |
| 3TC/DTG | 6 (11.1) | 1 (6.7) | 4 (8.9) | 9 (7.6) |
| 3TC/DTG+TDF+LPV/r | 0 (0.0) | 0 (0.0) | 1 (2.2) | 0 (0.0) |
| 3TC+EFV+TAF | 0 (0.0) | 0 (0.0) | 1 (2.2) | 0 (0.0) |
| 3TC + ABC + LPV/r | 0 (0.0) | 0 (0.0) | 0 (0.0) | 2 (1.7) |
| 3TC/DTG + TDF | 0 (0.0) | 0 (0.0) | 0 (0.0) | 2 (1.7) |
| 3TC + EFV + TDF | 36 (66.7) | 7 (46.7) | 26 (57.8) | 72 (61.0) |
| ANV/3TC/TDF | 0 (0.0) | 1 (6.7) | 0 (0.0) | 3 (2.5) |
| 3TC + NVP + TDF | 1 (1.9) | 0 (0.0) | 1 (2.2) | 1 (0.8%) |
| 3TC + TDF + LPV/r | 3 (5.6) | 0 (0.0) | 3 (6.7) | 6 (5.1) |
| AZT/3TC + EFV | 6 (11.1) | 3 (20.0) | 3 (6.7) | 14 (11.9) |
| AZT/3TC + LPV/r | 1 (1.9) | 2 (13.3) | 3 (6.7) | 5 (4.2) |
| AZT/3TC + NVP | 0 (0.0) | 0 (0.0) | 2 (4.4) | 1 (0.8) |
| BIC/FTC/TAF | 1 (1.9) | 1 (6.7) | 1 (2.2) | 3 (2.5) |
| **Inspection information (Median, IQR)** |  |  |  |  |
| CD4 lymphocyte count (cells/μL) | 488 (333, 610) | 520 (418, 641) | 446 (310, 582) | 479 (316, 680) |
| CD8 lymphocyte count (cells/μL) | 639.0 (495.0, 912.0) | 728 (525, 925) | 664 (460, 922) | 688. (330, 736) |
| HIV virus load (C/mL) |  |  |  |  |
| <50 | 51 (94.4) | 15 (100.0) | 41 (91.1) | 7 (5.9) |
| ≥50 | 3 (5.6) | 0 (0.0) | 4 (8.9) | 111 (94.1) |
| TG (mmol/L) | 1.24 (0.89, 1.96) | 1.32 (0.93, 1.73) | 1.44 (0.97, 2.45) | 1.43 (0.95, 2.26) |
| TC (mmol/L) | 4.67 (3.86, 5.02) | 4.93 (4.00, 5.11) | 4.83 (3.80, 5.33) | 4.52 (4.02, 5.05) |

Abbreviations: ABC, Abacavir; AZT, Azidothymidine; BIC, Bictegravir; DTG, Dolutegravir; EFV, Efavirenz; EVG/c, Elvitegravir/Cobicistat; FTC, Emtricitabine; LPV/r, Ritonavir‐boosted lopinavir; NVP, Nevirapine; TAF, Tenofovir Alafenamide; 3TC, Lamivudine; TDF, Tenofovir; TG, triglyceride; TC, total cholesterol

| **Risk factors** | **Breakthrough infection rate** | **Symptom incidence rate** |
| --- | --- | --- |
| **Time between infection to sampling (n, %)** |  |  |
| <60d | 100 | 24 (88.8) |
| 60-90d | 100 | 39 (90.6) |
| 90-120d | 100 | 76 (83.5) |
| 120-180d | 100 | 13 (81.2) |
| **Age (n, %)** |  |  |
| Young (18-40) | 90 (79.0) | 75 (65.7) |
| Middle-aged (41-60) | 69 (77.3) | 62 (69.6) |
| Elderly (>60) | 18 (62.0) | 15 (51.7) |
| **CD4 lymphocyte count (n, %)** |  |  |
| <350 | 50 (74.5) | 44 (65.6) |
| 350-500 | 44 (77.2) | 38 (66.6) |
| >500 | 83 (76.9) | 70 (64.8) |

**Table S2. Risk factors associated with breakthrough infection rate and symptom incidence among PLWH.**

**Figure S1**

**
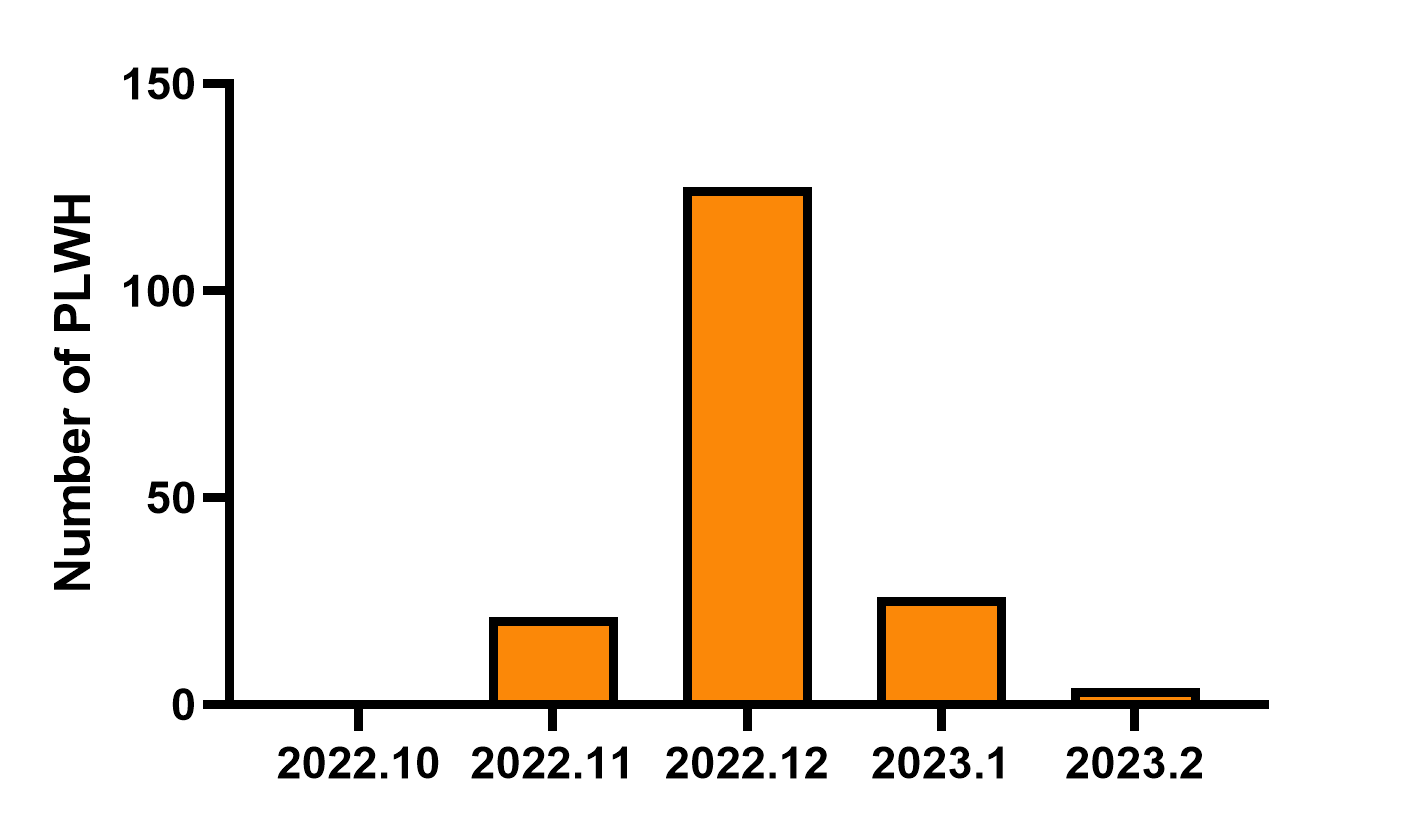
**
