# Supplementary material for: Humoral immunity and infection status of PLWH following vaccination after the BA.5/BF.7 wave
Source: Front Microbiol. 2026 Apr 1;17:1803277. doi: 10.3389/fmicb.2026.1803277 (PMC13079649; doi:10.3389/fmicb.2026.1803277)
Supplement: Supplementary file 2 [file Table_2.DOCX]

**Table S3 Questionnaire survey on SARS-CoV-2 infection among people living with HIV (PLWH)**

1. Your treatment number is ___.
2. Your gender is ___.
3. Man
4. Female
5. Your age is ___.

A. Below 18 years of age

B. 18-29 years old

C. 30-39 years old

D. 40-49 years old

E. 50-59 years old

F. 60-69 years old

G. 70 years old and above

1. Your vaccination status is ___.

A. Not vaccinated

B. One dose of vaccination has been administered

C. Two doses of vaccination have been given

D. Three doses of inoculation

E. Four doses of vaccination have been given

5. Have you had a new coronavirus infection since June 1, 2022?___

1. Not infected

B. Previously positive has turned negative

C. Currently testing positive

6. Whether or not it is tested by nucleic acid/antigen ___.

A. Nucleic acid test

B. Antigen self-testing

C. Both

7.The date of the first nucleic acid/antigen test is ___.

8. Whether a person living in the same household or a close contact is infected with the new crown ___.

A. Yes

B. No

9. Do you have any of the following symptoms? [Multiple choice question]___

A. No symptoms

B. Fever

C. Fatigue

D. Headache

E. Muscle aches

F. Sore throat

G. Nasal congestion

H. Runny nose

I. Cough

J. Expectoration

K. Taste and/or smell loss

L. Diarrhea

M. Emesis

N. Dyspnea

O. Other ____

10. Have you visited a healthcare facility for symptoms related to your new crown? ___

A. Failure to attend

B. Outpatient/Emergency

C. Square Cabin Hospital

D. Hospitalization

1. Do you have any other underlying conditions? [Multiple choice question]___

A. None

B. Hypertension

C. Hyperlipidemia

D. Coronary heart disease

E. Stroke

F. Diabetes

G. Chronic kidney disease

H. Chronic hepatitis

I. Chronic respiratory diseases (COPD, chronic bronchitis, bronchiectasis, interstitial pneumonia)

J. Severe opportunistic infections (PCP, viral pneumonia, tuberculosis, non-tuberculous mycobacterial infections, cytomegalovirus infections, herpes simplex, varicella-zoster virus, toxoplasmic encephalitis, fungal infections)

K. Cancer

L. Other ____
